# Effects of Modification on Properties of Wood Flour/PBAT Biocomposites

**DOI:** 10.3390/polym17050555

**Published:** 2025-02-20

**Authors:** Wangwang Yu, Rui Qiu, Wen Lei, Yong Chen

**Affiliations:** 1 School of Mechanical Engineering, Nanjing Vocational University of Industry Technology, Nanjing 210023, China; 2 College of Science, Nanjing Forestry University, Nanjing 210037, China; 3 Shanghai Hongrui Biotech. Co., Ltd., Shanghai 201199, China

**Keywords:** poly (butylene adipate-co-terephthalate), wood flour, composite, biodegradable, property

## Abstract

Wood flour (WF)-reinforced poly (butylene adipate-co-terephthalate) (PBAT) composites were successfully fabricated by injection-molding process after being mixed using an extruder. The effects of fiber modifications, including mercerization, acetylation, as well as coupling agent treatment on the properties of WF/PBAT composites, were studied. The results indicated that all the modifications increased the mechanical properties (e.g., tensile strength, tensile modulus, flexural strength, flexural modulus, elongation at break, and Charpy impact strength) of the composites. After modification, all the composites showed better interfacial bonding, hydrophobicity, and thermal properties compared to the untreated fiber composites; meanwhile, the moisture absorption test showed that all the modified fiber composites exhibited a much lower saturated water absorption rate than untreated ones. WF modification by addition of a coupling agent could improve the properties most obviously, except for the tensile strength, elongation at break, and saturated water absorption rate. By this modification, the tensile modulus, flexural strength, flexural modulus, impact strength, onset temperature during thermal degradation, degree of crystallinity, and water contact angle of the composite were 313.47 MPa, 20.55 MPa, 830.79 MPa, 16.01 kJ/cm^2^, 367.71 °C, 17.10%, and 101.8°, all increased from those of untreated composites by 17.95%, 30.73%, 87.52%, 35.79%, 61.49%, 25.67 °C, 89.16%, and 6.6°, respectively.

## 1. Introduction

With the growing concerns of environment protection and sustainable development, there is a growing interest in the development of degradable plastics as a substitute for petroleum-derived ones [1]. As mentioned, poly (butylene adipate-co-terephthalate) (PBAT) is currently one of the most popular biodegradable polymers. As an aliphatic-aromatic random copolyester prepared by chemical synthesis from fossil resources [2], PBAT can completely degrade within a few weeks by means of biological enzymes in the atmosphere [3] thanks to the presence of an unstable ester group in its main chain. PBAT has good processability and can be conveniently processed using the same methods as traditional plastics. The excellent flexibility gives PBAT great application potential in the fields of agricultural films, packaging industries, and medical devices [4]. However, it has also some undeniable downsides, including its high production costs and relatively low modulus and stiffness; these have limited its wider commercial utilization. To improve the properties and reduce the cost of PBAT while maintaining its degradable and compostable qualities, some modification techniques have been developed, such as blending PBAT with other polymers, nanofillers [3], and natural fibers [2]. Compared to the previous two, plant fibers are abundant, cheap, and easy to obtain, so they are more useful for blending with PBAT.

As one of the most popular biomass materials, wood flour (WF) is a byproduct of the wood industry; a large number of WF is produced every year, and it is cheap, light, biodegradable, and sustainable and has specific mass as well as low abrasion resistance [5]. Combining WF with petroleum-derived polymers such as polyethylene (PE) [6], polypropylene (PP) [7], and polyvinyl chloride (PVC) [8] has resulted in wood–plastic composites (WPC) that can be applied to produce numerous products, including decking, siding, floors, roofing, window frames, wall panels, and doors. Meanwhile, it can also be used to replace traditional construction materials such as wood, plastic, glass, and metal [9,10]. In recent years, production of sustainable materials has been made possible by combining WF with a biodegradable polymer, and much research has reported on this topic. For examples, polycaprolactone (PCL) is a typical biodegradable aliphatic polyester; it has good mechanical properties such as impact resistance and flexibility. Cintra et al. [5] developed new biodegradable composite materials from PCL and wood flour, and they found that the incorporation of WF made the materials more moisture-absorptive and was beneficial for the degradation by hydrolysis. As another kind of biodegradable polymer, poly (butylene succinate) (PBS) is produced through the polycondensation of 1,4-butanediol with succinic acid, and it has outstanding properties, such as high processability and strength as well as excellent thermal and chemical resistance, making it possible to be used to produce WPC with excellent properties. However, its melt flow ability and consequently the workability and quality of the resulting WPC would deteriorate after the addition of WF. To solve this problem, Park et al. [11] explored the effect of the application of kraft lignin (KL) and diphenylmethane diisocyante (pMDI) on the melt flow index (MFI) of the WF/PBS composites, and the results showed that the addition of KL increased the MFI, whereas pMDI addition decreased the MFI. As one of the two biopolymers that are expected to grow the most remarkably [12], poly (lactic acid) (PLA) has properties mostly like polystyrene; after appropriate modifications, it is possible to approximate the properties of polyethylene or polypropylene. It can be manufactured into various products, including transparent films and blowing preforms; in the case of PET bottles, incorporation of WF can reduce costs and increase application areas. Fabijański proved the feasibility of the processing and sample preparation process of WF/PLA composites using injection-molding technology. It was reported that composite products could be obtained only by using an injection-molding machine with proper parameters of the technological process; it was unnecessary to mix PLA and WF on an extruder [13]. Saeed et al. [14] prepared composites using a PBS/PLA blend as matrix and wood flour as reinforcement, and they found that the resin and the filler were not compatible due to the poor wettability and interfacial adhesion, but a coupling agent called Fusabond MB 100 D, i.e., maleic anhydride-grafted high-density polyethylene (HDPE) with a high graft level, could enhance the interaction between the blend and WF.

In our previous work, we found that the cost of PBAT could be reduced significantly when blending with WF; however, some of its physico-mechanical performances concerning its mechanical properties and thermal stability became poor [15]. For its wider applications, it is quite necessary to perform some modifications to improve the properties of the composites. The research presented in this article is a continuation of our previous work; in this work, three modification methods are introduced, and the comprehensive performances of WF/PBAT composites are enhanced obviously after modification, showing that WF/PBAT can be used in the manufacture of some products, such as package boxes, trays, and agricultural film.

## 2. Experimental

### 2.1. Materials and Reagents

PBAT in pellet form was purchased from Xinjiang Blue Ridge Tunhe Sci. & Tech. Co., Ltd., China (Changji, Xinjiang, China); WF, 80 mesh, was kindly supplied by Nanjing Dayuan Ecological Construction Group, China (Nanjing, Jiangsu, China); sodium hydroxide (NaOH, AR) and acetic anhydride ((CH_3_CO)_2_O, CP) were both purchased from Nanjing Chemicals Reagents Co., Ltd. (Shanghai, China); KH550 (H_2_N(CH_2_)_3_Si(OC_2_H_5_)_3_) was purchased from Shanghai Lingfeng Chemicals Reagents Co., Ltd. (Shanghai, China).

### 2.2. WF Modification

#### 2.2.1. Mercerization

WF was dried at 105 °C for 24 h, and then, the pre-dried WF was dipped in 5 wt.% NaOH solution at room temperature for 8 h. The sodium hydroxide solution to WF ratio was 20:1 in terms of weight percent (wt.%). After mercerization, alkali fibers were taken out from the solution and washed until the eluent was neutral; after that, they were dried at 105 °C to constant mass, and the obtained alkaline-treated WF was named A-WF.

**Figure 1:**
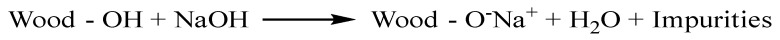
Schematic diagram of reaction mechanism for alkali treatment of WF [16].

The reaction mechanism is illustrated in Figure 1.

#### 2.2.2. Coupling Agent Treatment

A 95 wt.% ethanol solution was prepared by mixing ethanol and distilled water; next, KH550 was mixed well with the 95 wt.% ethanol solution in the ratio of 4:96 to form KH550 solution and allowed to stand for several hours. The pH of the KH550 solution was maintained at a value of around three to bring about the complete hydrolysis of the silane by the addition of acetic acid; then, dried wood flour was impregnated with the KH550 solution and mixed homogeneously, and after being maintained at room temperature for 12 h, the wood flour was taken out, washed many times with distilled water until the eluent was neutral, and dried at 105 °C to constant mass. The obtained KH550-treated WF was named C-WF.

**Figure 2:**
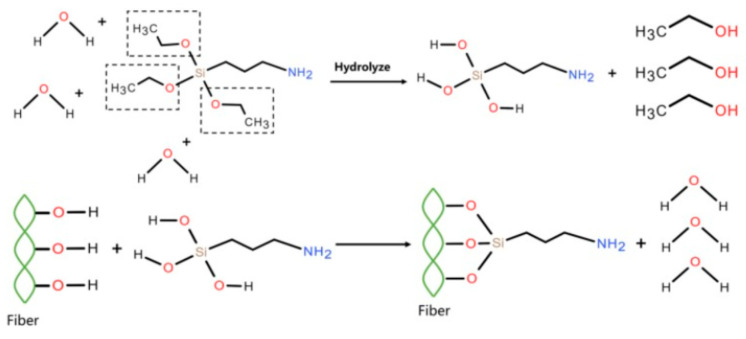
Schematic diagram of reaction mechanism for KH550 treatment of WF [17].

The reaction mechanism is illustrated in Figure 2.

#### 2.2.3. Acetylation

The acetylating solution was prepared by mixing 5 wt.% acetic anhydride with distilled water, and then, the dried A-WF was soaked in at 120 °C for 1.5 h; after modification, acetylated fibers were rinsed out in distilled water until the eluent was neutral, and finally, the wood flour was dried at 105 °C to constant mass. The obtained acetylated WF was named E-WF.

**Figure 3:**
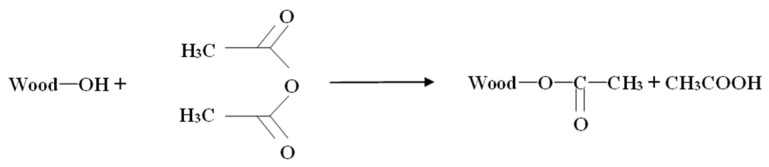
Schematic diagram of reaction mechanism for acetylation treatment of WF [18].

The reaction mechanism is illustrated in Figure 3.

### 2.3. Sample Preparation

Prior to processing, all the raw materials were treated at 105 °C to constant masses to remove any trace of moisture. Then, the dried wood flour and PBAT were weighed by 50:50 and added to a container and mixed well with a muddler at room temperature. Then, the wood flour and PBAT mixture was extruded and pelletized using a twin-screw extruder machine (SHJ-20, Nanjing Giant Machinery Co., Ltd., Nanjing, China). The extruder temperatures were between 105 °C and 120 °C, and the rotating speed of the screw was set at 105 r/min. Subsequently, the pellets were injection-molded using an electric injection-molding machine (CWI-90BV, Shanghai Jiwei Machinery Industry Co., Ltd., Shanghai, China) to obtain the samples for testing; the injection temperature was controlled in a range from 120 °C to 150 °C, and the injection pressure was kept at 140 MPa.

The samples containing WF, A-WF, C-WF, and E-WF were named WF/PBAT, A-WF/PBAT, C-WF/PBAT, and E-WF/PBAT, respectively.

### 2.4. Testing and Characterization

#### 2.4.1. Fourier-Transform Infrared Spectroscopy (FTIR) Analysis

Infrared spectra were obtained using a VERTEX 70 spectrometer (Bruker Optics, Ettlingen, Germany) equipped with a diamond crystal ATR reflection accessory. The WF, A-WF, C-WF, and E-WF samples were prepared with KBr powder as the background by mixing an approximate 1 wt.% of the wood flour. Spectra analysis was conducted with a resolution of 4 cm^−1^, with 32 scans obtained in the region between 4000 cm^−1^ and 500 cm^−1^. Before measurement, the background spectrum was recorded.

#### 2.4.2. Mechanical Characterization

A tensile test was conducted according to ASTM D 638-2010 [19] at a crosshead speed of 50 mm/min using a universal testing machine (E44.304, MTS Industrial Systems (China) Co., Ltd., Shenzhen, China) at room temperature, and the three-point flexural test was carried out according to ASTM D 790-2010 [20] using the same testing machine mentioned above at a crosshead speed of 5 mm/min and a span of 80 mm.

A dynamic Charpy impact test was conducted according to Chinese National Standard GB/T 1043.1-2008 [21] using a universal impact testing machine (XJC-25D, Chengde Precision Testing Machine Co., Ltd., Chengde, China).

The mean values and standard deviations of all mechanical properties represent an average of five samples.

#### 2.4.3. Scanning Electron Microscopy (SEM) Observation

Hitachi SU 8010 scanning electron microscopy (SEM, Hitachi Corporation, Tokyo, Japan) was used to obtain images of the composite fractured surfaces and to evaluate the effect of WF modification on the interface between fibers and the PBAT matrix. The observation was realized with an accelerating voltage of 3 kV. For a better resolution, each sample was covered with a thin layer of gold on its surface.

#### 2.4.4. Water Absorption Test

Five WF/PBAT, A-WF/PBAT, C-WF/PBAT, or E-WF/PBAT samples were immersed in a water bath at ambient temperature; removed at selected immersion times; wiped using tissue paper; and then weighed at room temperature on a balance with a precision of 0.1 mg. The water absorption rate $w_{a}$ (%) at time t was determined by Equation (1):

(1) $$w_{a}(\%)=\frac{m_{t}-m_{0}}{m_{0}}\times 100$$

where $m_{0}$ and $m_{t}$ represent, respectively, the weight of the samples before and after the test. The average percentage increase in $w_{a}$ (%) for the samples was calculated from the values of several consecutive measurements that showed a negligible variation of water absorption.

#### 2.4.5. Thermal Stability Assessment

A thermal gravimetric analyzer (TG209F1, NETZSCH Gerätebau GmbH, Selb, Germany) was used to evaluate thermal stability of WF/PBAT, A-WF/PBAT, C-WF/PBAT, and E-WF/PBAT samples. The measurements were performed under nitrogen atmosphere. The experimental parameters were as follows: sample dosage, 3–5 mg; heating temperature, 30–600 °C; heating rate, 30 °C/min; and argon flow rate, 20 mL/min. Thermogravimetric (TG) curves and derivative thermogravimetric (DTG) curves were recorded and analyzed using NETZSCH software (TG209 F1 Libra). The 5% weigh loss temperature (T_i_) collected from the TG data and the maximum degradation temperature (T_p_) collected from the derivative DTG data were used to distinguish differences arising from WF modifications.

#### 2.4.6. Melting and Crystallization Behavior Analysis

A differential scanning calorimetry (DSC) apparatus, model NETZSCH DSC214 (NETZSCH-Gerätebau GmbH, Selb, Germany), was used to determine the melting and crystallization behavior of the samples. DSC tests were run using 3–5 mg samples under the following cycles: heating from 20 °C to 220 °C at a heating rate of 10 K/min, holding at 220 °C for 5 min, then cooling down to room temperature, and finally reheating to 220 °C. All the heating and cooling cycles were completed under nitrogen atmosphere, using 20 mL/min of flow rate.

The melt temperature (T_m_), cold crystallization temperature (T_cc_), fusion enthalpy (Δ*H_m_*), and cold recrystallization enthalpy ($\Delta H_{cc}$) were determined based on the DSC test. The degree of crystallinity ($\chi_{c}$) was calculated using the following equation:

(2) $$x_{c}=\frac{\left|\Delta H_{m}+\Delta H_{cc}\right|}{\omega \Delta H^{\theta }}\times 100\%$$

where $\Delta H^{\theta }$ is the theoretical enthalpy of a fully crystallized PBAT with a value of 114 J/g [22]. ω stands for the PBAT mass fraction obtained from the TGA and normalizes the result considering the percentage of WF in the material.

#### 2.4.7. Wettability Testing

The surface wettability test was conducted at room temperature by contact angle measurements with a contact angle instrument (DSA100; KRÜSS GmbH, Borsteler Chaussee, Hamburg, Germany) and the corresponding software for this device. The contact angle (θ) of distilled water drops on the surface of each specimen was measured by dropping 5 µL droplet of distilled water onto the surface, maintaining for 15 s, and then reading the θ values at various points. The mean value and standard deviation of the contact angle represent an average of five samples.

## 3. Results and Discussion

### 3.1. FTIR Analysis

**Figure 4:**
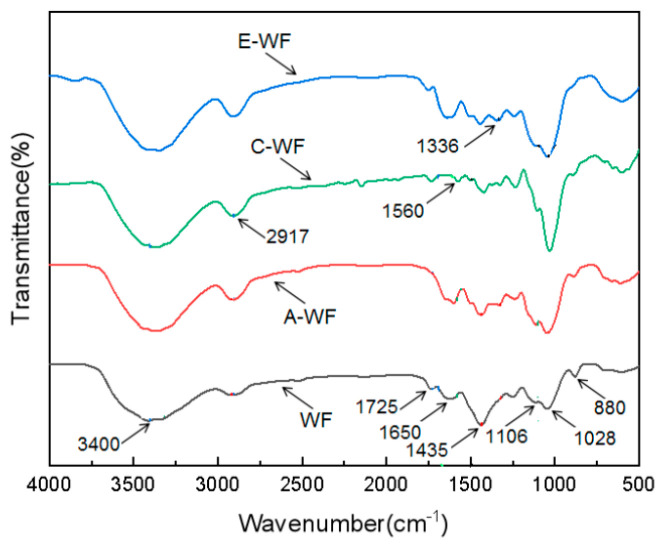
FTIR spectrogram of unmodified/modified WF.

Figure 4 presents the Fourier-transform infrared (FTIR) spectra of WF before and after treatment. For each sample, there was a characteristic wide band at around 3400 cm^−1^ that corresponded to the stretching vibrations of the -OH group and hydrogen bonds of hydroxyl groups [23], a peak near 1435 cm^−1^ related to the symmetrical bending of the -CH_2_ group present in cellulose [23], and a peak at 1028 cm^−1^ attributed to the -C-O stretching vibrations of the hydroxyl groups and ethers in cellulose [23]. The peaks at around 1106 cm^−1^ and 880 cm^−1^ were associated with the C-O-C pyranose ring stretching vibration and β-glycosidic linkages of glucose rings, respectively. These two absorption peaks were found in all the samples, indicating that the structure of cellulose in WF was not affected by chemical treatments [24].

In the spectra recorded for unmodified WF, the band at around 1725 cm^−1^ was attributed to the stretching vibrations of the carboxyl group in lignin or the ester group in hemicellulose [23,24], which was the characteristic peak of hemicellulose and lignin [25]. After alkali treatment, part of the hemicellulose and lignin in the wood fiber was effectively removed, resulting in attenuation of the characteristic peak [17,25]. When A-WF was treated by acetylation, the peak intensity at around 1725 cm^−1^ significantly increased once again; in addition, a new absorbance peak at 1336 cm^−1^ appeared, which was assumed to have resulted from the carboxyl group in acetic anhydride, meaning that a reaction between WF and acetic anhydride had occurred in E-WF. Compared with WF, C-WF showed an increased peak intensity at 2917 cm^−1^ due to the introduction of the CH_2_ group of KH550 and a new characteristic absorption peak at 1560 cm^−1^, corresponding to the bending vibration peak of -NH_2_ groups in the silane coupling agent [17]; meanwhile, the intensity of the absorbance at about 1650 cm^−1^ (H-O-H bending) decreased significantly due to the loss of some water molecules produced by the reaction between hydrolyzed KH550 and WF [26]. These results show that KH550 was successfully imported to the surfaces of wood fibers, which agrees with the results reported by Zhang et al. [17] and Liu et al. [27].

### 3.2. Mechanical Properties Analysis

When PBAT is compounded with wood flour by melt-blending process, lots of hydrogen bonds will be formed between polar hydroxyl groups (-OH) from cellulose and hemicellulose in WF and ester groups (-COO-) in PBAT, which become the main force between the reinforcement and the matrix. The wood flour particles are embedded in the PBAT matrix, forming a physically entangled structure that enables WF/PBAT composite’s proper mechanical performance. When WF is treated, the interfacial bonding between WF and PBAT may be affected, leading to the changes in mechanical properties.

**Figure 5:**
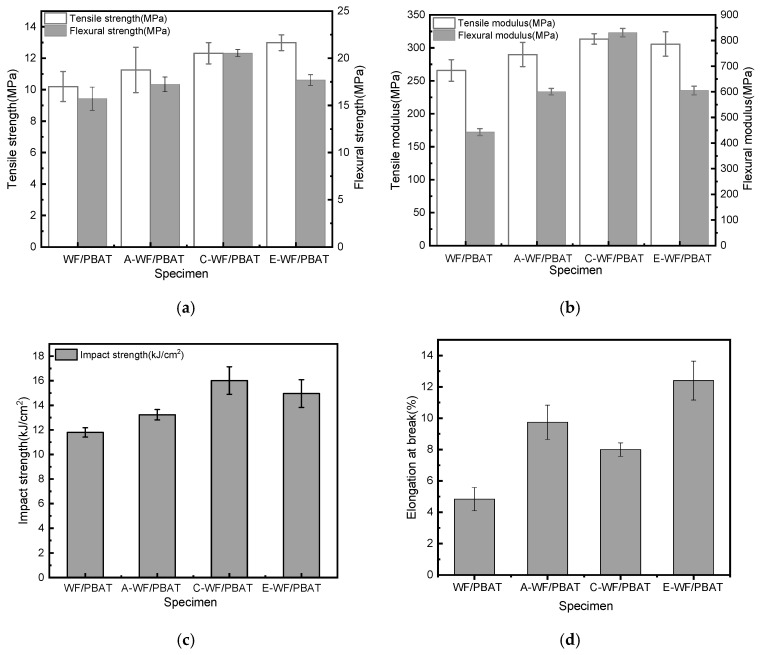
Mechanical properties of composites with different modification methods: (**a**) tensile and flexural strengths; (**b**) tensile and flexural moduli; (**c**) impact strength; (**d**) elongation at break.

The effects of WF modification on the mechanical properties of the composites are shown in Figure 5. The tensile strength and modulus, flexural strength and modulus, Charpy impact strength, as well as elongation at break of WF/PBAT were found to be 10.20 MPa, 265.77 MPa, 15.72 MPa, 443.04 MPa, 11.79 kJ/m^2^, and 4.83%, respectively. After WF modification, all these properties were modified to different extents, among which, alkali treatment increased the values by 10.39%, 9.07%, 9.61%, 35.54%, 12.21%, and 101.66%, respectively; after acetylation, the increases from those of WF/PBAT were 27.35%, 15.07%, 12.60%, 36.62%, 26.80%, and 156.94%. When WF was treated with KH550, C-WF/PBAT had a tensile strength, tensile modulus, flexural strength, flexural modulus, Charpy impact strength, and elongation at break of 12.32 MPa, 313.47 MPa, 20.55 MPa, 830.79 MPa, 16.01 kJ/m^2^, and 7.80%. In this case, the mechanical properties were improved from the unmodified samples by 20.78%, 17.95%, 30.73%, 87.52%, 35.79%, and 61.49%, respectively.

One of the reasons for the better mechanical properties after alkali modification was that the fiber thickness was reduced [28]: Some of the chemical substances, such as hemicellulose and lignin in WF, were removed, as evidenced from FTIR spectra analysis, making the original smooth wood fiber surface rough [29] and leading to pronounced fibrillation [6]. As a result, more chemical bonding structures were formed, and the bonding between WF and PBAT was enhanced, and this improved interfacial bonding was also found in the SEM observations shown in Section 3.3. Meanwhile, the amorphous zone was reduced in the fiber with the removal of amorphous lignin from WF, and a more compact arrangement of cellulose was obtained.

Compared to A-WF/PBAT, E-WF/PBAT showed further improved mechanical properties. This improvement might be also explained by the importance of the interaction between WF and PBAT. As discussed above, mercerization enhanced the interfacial bonding in the composite; when being acetylated, A-WF reacts more easily with the carboxyl groups of the acetic anhydride during esterification [6]. The hydroxyl groups in WF are thus replaced with ester groups, which are likely to promote fiber–polymer interaction and therefore improve the compatibilization of the fibers with the PBAT in the composite. In addition, acetylation may also roughen the surface of the fiber and create many voids, leading to greater adhesion between fibers and polymer [30], so that the NaOH and acetic anhydride synergistic modification can maximize the mechanical properties of the composites.

When KH550 was applied to treat WF, the ethoxy groups in its structure hydrolyzed to form silanol, which reacted with the hydroxyl groups on the surface of the wood fiber to form an alkoxy structure [17] and hydrogen bonds [31], and some low-polar radicals (CH_2_=CH-, NH_2_-CH_2_-CH_2_-CH_2_-) were imported onto the surface of WF [27]. Meanwhile, the γ-aminopropyl group in KH550 was compatible with PBAT. Consequently, KH550 acted as a bridge between the PBAT and wood fiber. The bridge effect contributed to enhancing the performance of the composite [17].

### 3.3. SEM Analysis

**Figure 6:**
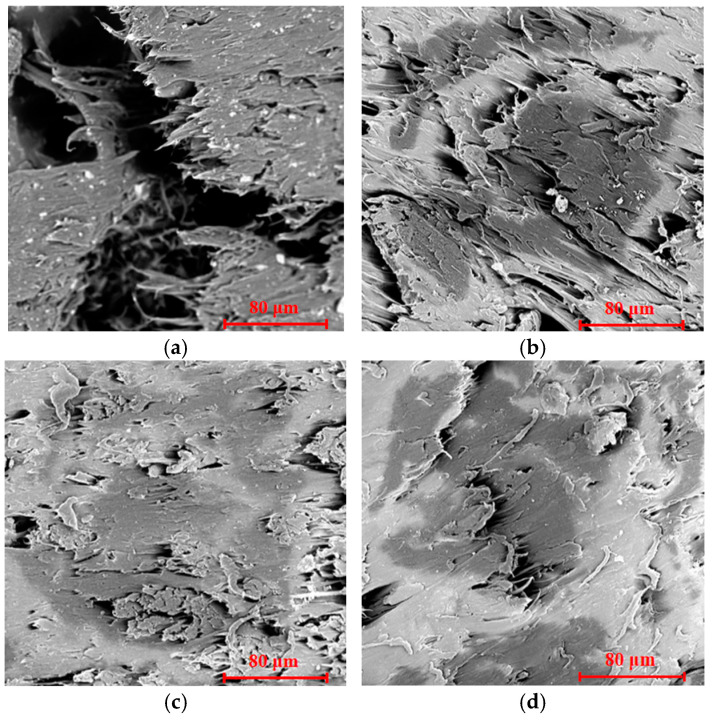
SEM images of composites: (**a**) WF/PBAT; (**b**) A-WF/PBAT; (**c**) C-WF/PBAT; (**d**) E-WF/PBAT.

The SEM characterization revealed a significant change in the morphologies of the composites before and after WF modification, as depicted in Figure 6. The micrographs in Figure 6a show poor interfacial compatibility between WF and PBAT; in this situation, WF was not wrapped tightly with PBAT and presented a disorderly state inside the resin. Many fiber agglomerations and voids (shaded part of the figure) were present in the composites. This similar phenomenon was also found in some other composites, such as wood fiber/polyurethane [17], micro-nano rice husk/polylactic acid [26], hemp fiber/polyester [32], and agave fiber/PVC [6] composites. The poor interaction and low compatibility between WF and PBAT led to interface debonding and stress concentration when the composite was stressed; therefore, the mechanical properties and water resistance of composites were decreased, as described in Section 3.2 and the following Section 3.4, respectively. Figure 6b–d reveal that treating the fibers with alkali, acylation, or coupling agent made the fracture surfaces much more homogeneous. WF was distributed more evenly in the matrix and was hard to peel out from the PBAT matrix. The good interfacial compatibility was still maintained upon breaking, which was the most pronounced for C-WF/PBAT (Figure 6c). After KH550 modification, the dispersed-phase C-WF of the composites became smaller and was distributed in the continuous phase of PBAT uniformly. The interface between the two phases became blurred, and all the fibers were almost wrapped together with the matrix; interfacial debonding could hardly be found, and this morphological change might be related to the removal of the extractives in WF and the dissolution of lignin and hemicelluloses from the outer surface of fibers and also the interaction between the fiber and the modifier, indicating that WF modification improved the interfacial bonding between the reinforcement and the matrix [31,33]. Opposite to those of the pristine WF/PBAT, the enhanced interfacial bonding in the modified composites observed by SEM thus improved the mechanical performance and reduced water absorption, which is consistent with the results depicted in Section 3.2 and the following Section 3.4, respectively.

### 3.4. Water Absorption Analysis

**Figure 7:**
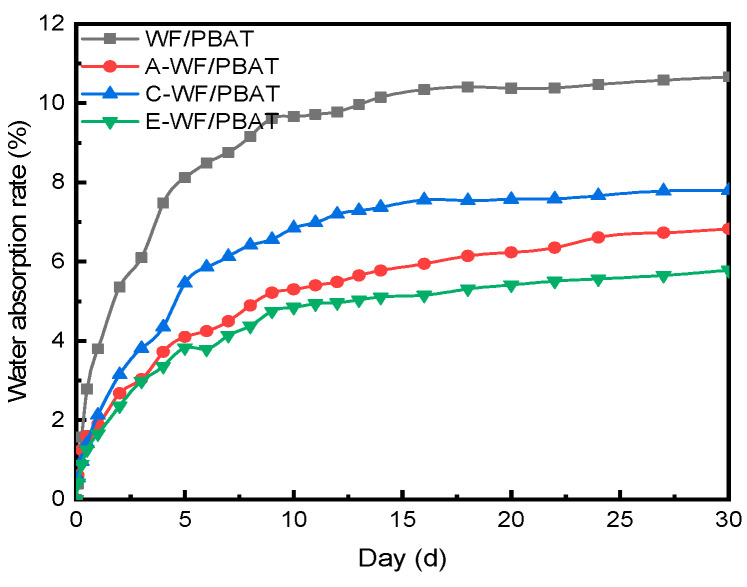
Water absorption curves of composites with different modification methods.

Figure 7 demonstrates the effects of soaking time on the water absorption of each composite. The data showed large increases in absorption for all the samples up to 5 days, with very slow increases at times beyond 5 days. A similar trend was also reported on some other wood fiber-reinforced composites [34]. Compared to WF/PBAT, all the modified composites absorbed much less water at any immersion stage after 5 days, especially for E-WF/PBAT, where the reduction in water absorption was the most significant: it only absorbed water by 5.78% after soaking in water for 30 days, which is a reduction from that of WF/PBAT by 45.79%. Some of the literature has analyzed the mechanisms for the reduction in water absorption of natural fiber-reinforced plastics after fiber modifications; for example, Lynda Chelali et al. [6] thought that alkali treatment could reduce the water absorption of *Agave americana* fibers/PVC composites mainly due to the removal of a large part of the hemicellulose. When investigating the effects of acetylation treatment on the properties of kenaf/starch composites, Jung et al. [30] reported that fiber treatment with acetic anhydride formed acetyl groups through the condensation of -OH groups, which hydrophobized the fiber and reduced its absorbency. These investigations provide reasonable explanations for the decreased water absorption by the modified WF/PBAT composites when compared to the unmodified ones. It is worth noting that C-WF/PBAT had the most homogeneous fracture surface among all the composites, as revealed by the SEM observation shown in Section 3.3, while E-WF/PBAT absorbed the least water. This inconsistency between the results of the SEM observation and water absorption test was attributed the sophisticated water absorption mechanism; besides interfacial structure, both the fiber and the matrix themselves also had great effects on the water uptake ability of a composite. 

### 3.5. Thermal Stability Analysis

**Figure 8:**
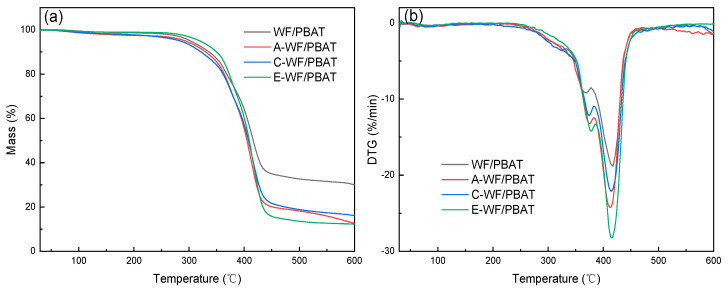
Thermogravimetric analysis of composites with different modification methods: (**a**) TG curves; (**b**) DTG curves.

The decomposition processes and the thermal stabilities of the composites before and after WF modification were investigated using a TGA. Figure 8a,b illustrate the TGA and DTG curves of the composites. It can be observed that there was no obvious difference in the decomposition processes of the composites, showing that WF modification did not change the thermal decomposition mechanism of the composites. Further research indicated that there was a minor mass loss for each sample before 150 °C, which was attributed to the evaporation of moisture or low volatile impurities [35,36]. The main decomposition was found to occur between 200 °C and 500 °C, and the detailed results are tabulated in Table 1. At this stage, all the composites started to degrade at approximately 230 °C, corresponding to the degradation of WF [35]. From Table 1, it can be found that the T_i_ values of A-WF/PBAT, C-WF/PBAT, and E-WF/PBAT were 364.53 °C, 367.71 °C, and 365.23 °C, respectively, and all the modified composites exhibited a much higher T_i_ than WF/PBAT. In addition, after modification, the T_p,1_ of the composite was accordingly increased, meaning that the incorporation of modified WF into PBAT improved thermal behavior as compared to the untreated composites. This result is coherent with the research findings on other natural fibers [37] and their reinforced degradable polymers [38].

### 3.6. Crystallization and Melting Behavior Analysis

**Figure 9:**
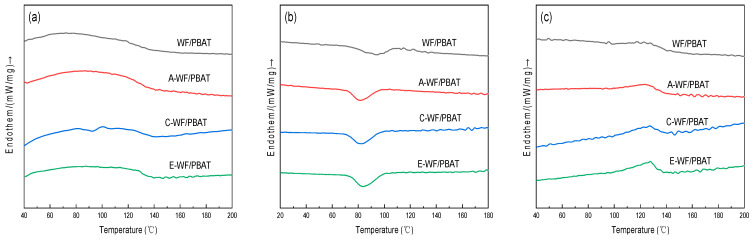
DSC analyses of different composites: (**a**) first heating; (**b**) cooling; (**c**) second heating.

The DSC heating and cooling thermograms of WF/PBAT, A-WF/PBAT, C-WF/PBAT, and E-WF/PBAT are exhibited in Figure 9 and summarized in Table 2. The crystallization procedure of a polymer usually consisted of two steps: nucleation and crystal growth. A lower T_cc_ means that it is easier for the polymer to carry out nucleation and also that the growth finishes at a faster speed; consequently, such a polymer would behave a better crystallization ability [39]. As shown in Table 2, the T_cc_ of WF/PBAT was 94.7 °C, and this temperature was decreased by 12.1 °C, 12.3 °C, and 10.9 °C for the composite with A-WF, C-WF, or E-WF, respectively, indicating that the modified composites could more easily crystallize, which is in accordance with the results of the calculated crystallinity also recorded in the table. The crystallinity of WF/PBAT was 9.04%, while those of A-WF/PBAT, C-WF/PBAT, and E-WF/PBAT composites increased significantly to 15.60%, 17.10%, and 15.79%, respectively, which can be attributed to the nucleation effect of modified WF [40]. Moreover, both the C-WF/PBAT and E-WF/PBAT composites had increased Xc% compared to the alkali treatment for the WF/PBAT composites.

When exploring the effect of fiber treatment on the melting behaviors of a nonwoven, unidirectional, matted banana empty-fruit-bunch fiber/ PP composite, Zaman et al. [40] found that both alkali and acetylation treatments increased its melting temperature (T_m_) and enthalpy of fusion (ΔH_m_) owing to the improved interfacial interaction of the fiber with the polymer matrix; similarly, KH550 treatment also increased the T_m_ of a micro-nano rice husk/polylactic acid composite, as reported by Sun et al. [26]. The same changing trends in T_m_ and ΔH_m_ were observed in this study, in which the T_m_ and ΔH_m_ of virgin WF/PBAT were 123.0 °C and 5.15 J/g, respectively (Table 2), after WF modification, and by acylation and coupling agent treatments especially, they both rose remarkably; this increase in ΔH_m_ indicated the increase in energy required to melt the composite as compared with WF/PBAT.

### 3.7. Hydrophobicity Analysis

**Figure 10:**
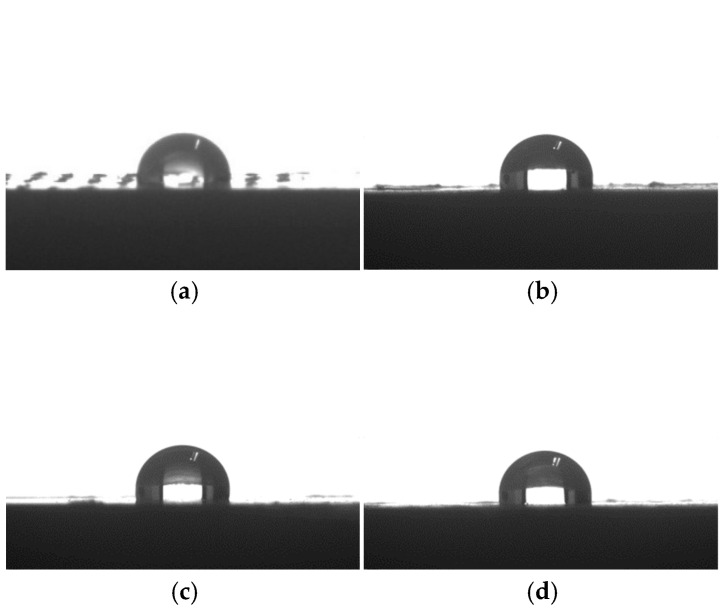
Surface contact angle topographies of different composites: (**a**) WF/PBAT; (**b**) A-WF/PBAT; (**c**) C-WF/PBAT; (**d**) E-WF/PBAT.

As one of the most important interface characteristics, the surface wettability of the composite, is determined by both its microstructures and the surface chemical compositions [41]; in other words, the surface wettability has a strong relationship with its properties. The shapes of the water droplets on the surfaces of the samples are shown in Figure 10. It can be observed that the surface wettability of the composites was different according to the different modification methods. The water contact angle of WF/PBAT was 95.2°, and after alkali, KH550, and acylation modifications, the surface contact angles of the composites increased accordingly to 97.1°, 101.8°, and 99.9°, indicating that the hydrophobicity of the modified composites increased. Moreover, the order of the water contact angle was C-WF/PBAT > E-WF/PBAT > A-WF/PBAT, and the increase in the contact angle of the C-WF/PBAT composite was more significant than that in A-WF/PBAT and E-WF composites. Generally, a material with a water contact angle greater than 90° is thought to be hydrophobic, and a greater contact angle demonstrates its greater hydrophobicity. For WF/PBAT, the contact angle of 95.2° showed that WF with many hydrophilic hydroxyl groups had been wrapped together with PBAT to some extent. After WF modifications, the hydrophilicity of WF was reduced, leading to the increase in the contact angle of the composite. Notably, the greatest contact angle of C-WF/PBAT in all the samples meant that the hydrophobicity of WF can be improved the most obviously. The reason for this could be that the hydrolysis of KH550 generated silanol, which reacted with the hydroxyl groups on the surface of the wood fiber to form an alkoxyl structure, dehydrated to form a chemical bond attached to the fiber [17], and formed a superficial coating with a low-polar radical, leading to an increase in the contact angle [27].

## 4. Conclusions

Incorporation of WF into PBAT to form composites would reduce the cost of the resin effectively, but some physico-mechanical properties would become poor. These issues were reported in our previous work. The present work is a follow-up to the preceding study. In this work, WF was treated by three methods, i.e., alkali treatment, acetylation treatment, and coupling agent treatment, and the effects of the WF modifications on the physico-mechanical properties of WF/PBAT composites were investigated. The following conclusions can be drawn from the experimental results:

(1) For the mechanical measurements, the treated composite systems showed improvements in all the properties, including tensile properties (tensile strength, tensile modulus, and elongation at break), flexural properties (flexural strength and modulus), and impact properties, among which the coupling agent treatment gave the composite the greatest tensile modulus, elongation at break, flexural strength and modulus, as well as impact strength;
(2) For the thermal behaviors, the modifications did not change the degradation and crystallization mechanisms but improved the thermal stability and crystallinity of the samples. Among all the composites, C-WF/PBAT had the greatest T_i_ and T_p,1_, showing that it was the most thermally stable. Meanwhile, it had the greatest crystallinity;
(3) Treated fibers were wrapped more tightly by PBAT when the composites were fractured. The modified composites, especially C-WF/PBAT, had much more homogeneous morphologies of fractured surfaces than untreated ones. The morphological observation supported the improved mechanical properties and reduced water absorption of modified WF/PBAT compared to pristine WF/PBAT;
(4) After WF modifications, the hydrophobicity of WF/PBAT was enhanced, especially for C-WF/PBAT: it had the greatest water contact angle of 101.8°, while that of pristine WF/PBAT was only 95.2°. In addition, WF treatments obviously reduced the water absorption by the composites.

In summary, the introduced modifications, especially treating WF with a coupling agent, had positive effects on the mechanical, thermal, and melting properties of the specimens; meanwhile, the surface hydrophobicity of the composite was improved, and less water was absorbed by the sample. All these improved properties make it possible for WF/PBAT composite to be used more widely.

## Figures and Tables

**Table 1:** Thermogravimetric analysis of composites with different modification methods.

Sample	T_i_ (°C)	T_p,1_ (°C)	T_p,2_ (°C)	T_f_ (°C)
WF/PBAT	342.04	368.98	415.14	440.82
A-WF/PBAT	364.53	376.12	412.44	433.01
C-WF/PBAT	367.71	378.52	415.22	440.63
E-WF/PBAT	365.23	373.74	414.31	438.35

**Table 2:** Thermal properties of composites with different modification methods calculated based on standardized DSC data.

Sample	T_cc_/°C	ΔH_c_ (J/g)	T_m_/°C	ΔH_m_ (J/g)	X_c_/%
WF/PBAT	94.7	−6.19	123.0	5.15	9.04
A-WF/PBAT	82.6	−7.586	123.5	8.89	15.60
C-WF/PBAT	82.4	−9.12	127.1	9.75	17.10
E-WF/PBAT	83.8	−9.025	127.9	9.00	15.79

## Data Availability

The original contributions presented in this study are included in the article. Further inquiries can be directed to the corresponding author.
